# Terpenoid-enriched *Curcuma wenyujin* nanovesicles for suppressing inflammation and restoring lipid homeostasis in MASH

**DOI:** 10.20517/evcna.2025.181

**Published:** 2026-06-30

**Authors:** Lin Liu, Jiale Niu, Yulong Sun, Zhuoyan He, Ziming Jiao, Guoen Li, Ganglin Wang, Fangjun Luo, Wei Li

**Affiliations:** ^1^Key Laboratory of Laboratory Medicine, Ministry of Education of China, Zhejiang Provincial Key Laboratory of Medical Genetics, School of Laboratory Medicine and Life Sciences, Wenzhou Medical University, Wenzhou 325035, Zhejiang, China.; ^2^Department of Clinical Laboratory, Zhuji People’s Hospital of Zhejiang Province, Zhuji 311800, Zhejiang, China.; ^#^These authors contributed equally to this work.

**Keywords:** *Curcuma wenyujin*, nanovesicles, terpenoid, inflammation, lipid homeostasis, MASH

## Abstract

**Aim:** This study investigates the potential of *Curcuma wenyujin*-derived nanovesicles (CW-DNVs) to ameliorate metabolic dysfunction-associated steatohepatitis (MASH) and explores their underlying mechanism, focusing on hepatic macrophage accumulation and the regulation of lipid metabolism.

**Methods:** CW-DNVs were isolated via ultracentrifugation and sucrose gradient purification, and their physicochemical properties, cellular uptake, and *in vivo* biodistribution were characterized. Anti-inflammatory and lipid-lowering effects were evaluated in liver macrophages, hepatocytes, and a high-fat diet (HFD)-induced MASH mouse model. Lipidomic, small-molecule, and small RNA (sRNA) cargoes were analyzed by liquid chromatography-tandem mass spectrometry (LC-MS/MS) and RNA sequencing.

**Results:** CW-DNVs were spherical (~211 nm diameter), had a zeta potential of -27.4 mV, and were enriched in lipids, proteins, sRNAs, and terpenoids like well-known bioactive curcumenol and germacrone. Following intraperitoneal injection, they preferentially accumulated in Kupffer cells and were cleared within 7 days. In HFD-fed mice, CW-DNVs reduced body weight gain, hepatic steatosis, serum aspartate aminotransferase (AST), alanine aminotransferase (ALT), triglycerides (TG), total cholesterol (TC), and hepatic interleukin (IL)-6 levels. Mechanistically, they upregulated peroxisome proliferator-activated receptor gamma coactivator 1-alpha (PGC-1α) and microsomal triglyceride transfer protein (MTTP), and downregulated fatty acid synthase (FASN), promoting lipid oxidation and export. *In vitro*, CW-DNVs suppressed lipopolysaccharide-activated IL-6, IL-1β, and tumor necrosis factor-alpha (TNF-α) in macrophages and reduced oleic acid-induced lipid accumulation in hepatocytes. sRNA sequencing identified predominantly rRNA-derived fragments (not canonical miRNAs); however, anti-inflammatory activity was primarily attributed to terpenoid components.

**Conclusion:** CW-DNVs exert dual functionality in modulating macrophage inflammation and lipid metabolism. Loaded with bioactive terpenoids, they represent an effective natural nanoplatform for MASH therapy.

## INTRODUCTION

Metabolic dysfunction-associated fatty liver disease (MAFLD) has become the most common chronic liver condition globally^[1,2]^. MAFLD encompasses a pathological spectrum that begins with hepatic steatosis, progresses to metabolic dysfunction-associated steatohepatitis (MASH), and may advance to fibrosis, cirrhosis, or even hepatocellular carcinoma^[3,4]^. As of 2025, two drugs have received FDA (Food and Drug Administration) approval for these conditions: resmetirom (Rezdiffra), the first-ever treatment, approved in 2024 for noncirrhotic MASH and moderate-to-advanced fibrosis, and semaglutide (Wegovy), which received accelerated approval in August 2025 for MASH^[5-8]^. These milestones highlight both progress and ongoing challenges in MASH therapeutics.

The complex pathogenesis of MASH involves multiple interconnected mechanisms, including dysregulated lipid metabolism and chronic inflammation^[9,10]^. Within this framework, activated hepatic macrophages, particularly Kupffer cells, serve as major drivers of disease progression by promoting the release of interleukin (IL)-1β, tumor necrosis factor-alpha (TNF-α), and IL-6. These cytokines perpetuate hepatocyte injury, promote steatosis, and foster the progression toward MASH^[11,12]^.

Plant-derived nanovesicles (PDNVs) have attracted considerable attention as novel bioactive entities with significant therapeutic potential^[13]^. These naturally occurring nanovesicles, isolated from various edible plants and traditional medicinal herbs, have demonstrated remarkable stability, biocompatibility, and the ability to mediate inter-species communication^[14,15]^. They encapsulate diverse bioactive molecules, including lipids, proteins, microRNAs (miRNAs), and small-molecule compounds, which collectively contribute to their pharmacological effects^[16,17]^. Notably, they are emerging modulators in inflammatory responses and metabolic pathways. For instance, ginger-derived nanovesicles have been shown to ameliorate osteoarthritis through the nuclear factor erythroid 2-related factor 2 (Nrf2) signaling pathway^[18]^, attenuate inflammatory bowel disease by inhibiting pro-inflammatory cytokines^[19]^, protect against alcohol-induced liver damage^[20]^, and prevent high-fat diet (HFD)-induced insulin resistance through forkhead box protein A2 (Foxa2)^[21]^. In contrast, grape-derived nanovesicles protect the skin from ultraviolet (UV) light-induced damage^[22]^. PDNVs therefore represent a promising, yet largely unexplored, resource for developing innovative therapeutics for inflammatory and metabolic disorders^[23-25]^.

Belonging to the Zingiberaceae family, *Curcuma wenyujin* (*C. wenyujin*) is a traditional Chinese herb that has long been used to treat diseases such as liver fibrosis^[26]^, jaundice^[27]^, and various inflammatory disorders^[28]^. Modern pharmacological studies have identified a rich array of bioactive compounds, including curdione, curcumenol, and germacrone, which exhibit well-documented anti-inflammatory and hepatoprotective properties^[29]^. Despite the rich bioactive composition of *C. wenyujin*, the potential therapeutic applications of its naturally occurring nanoparticles remain largely unexplored.

In this study, we isolated *Curcuma wenyujin*-derived nanovesicles (CW-DNVs) and characterized their physical properties and complex biomolecular composition. Subsequent experiments in an HFD-induced MASH mouse model revealed that CW-DNVs exerted dual regulatory effects on lipid metabolism and inflammation (Patent No: ZL 2023 1 0302280.0), with key small-molecule constituents mediating their core anti-inflammatory activity. These findings not only position CW-DNVs as promising agents against MASH but also highlight the potential of traditional herbal medicine to advance breakthroughs in nanomedicine.

## METHODS

### Isolation and characterization of plant-derived nanoparticles

#### Isolation of CW-DNVs

Selected rhizomes of *C. wenyujin* were 8-10 months old, weighing 200-300 g, and were sampled from November to January each year. Peeled, fresh *C. wenyujin* rhizomes were mixed with pre-cooled phosphate-buffered saline (PBS) at a 1:2 (w/v) ratio. The mixture was homogenized using a Blentec® homogenizer (Germany) with six 30-s cycles, followed by 30 s of rest. CW-DNVs were isolated from the homogenates using an established protocol combining differential ultracentrifugation with sucrose density gradient centrifugation^[13]^. Briefly, homogenates were subjected to sequential low-speed centrifugations (1,000 × *g* to 10,000 × *g*) to remove debris. The resulting supernatant was ultracentrifuged at 150,000 × *g* for 90 min. The pellet was resuspended in PBS and subsequently applied to the top of a discontinuous sucrose gradient containing layers of 8%, 30%, 45%, and 60% (w/v) sucrose. After a second ultracentrifugation (150,000 × *g* for 90 min), bands 1 (8%-30% interface) and 2 (30%-45% interface) were collected, washed in PBS to remove residual sucrose, and the final pellets were designated as CW-DNVs-1 and 2.

#### Transmission electron microscopy

CW-DNVs resuspended in PBS were applied to carbon-coated copper grids (10 μL/grid) and incubated for 3 min. The grids were negatively stained with phosphotungstic acid (2.5%, w/v) for 60 s, rinsed twice with PBS, air-dried, and then examined with a transmission electron microscope (JEOL Ltd., Tokyo, Japan) at 120 kV.

#### Nanoparticle tracking analysis

CW-DNVs were diluted in PBS to a concentration of 10^7^-10^9^ particles/mL and analyzed using a NanoSight (Malvern Instruments, Malvern, UK). Three 60-s measurements per sample were recorded, with particle concentration and size determined using nanoparticle tracking analysis (NTA) Software.

#### Zeta potential measurement

CW-DNV pellets were gently resuspended in PBS and the resulting suspension carefully transferred into a folded capillary cell. Triplicate zeta potential measurements were performed for each preparation at 25 °C.

### Characterization of CW-DNV cargo

#### Lipidomic profiling

An appropriate amount of CW-DNVs was mixed with 200 μL of PBS and 20 μL of internal lipid standard mixture. After brief vortexing, 800 μL of methyl *tert*-butyl ether and 240 μL of pre-chilled methanol were added and mixed by vortexing. Following a 20-min sonication in a 4 °C water bath, the mixture was left to stand for 30 min. The mixture was then centrifuged at 14,000 × *g* for 15 min at 4 °C, and the resulting upper organic phase was carefully transferred to a fresh tube and dried under nitrogen. The residue was reconstituted in 90% isopropanol/acetonitrile (v/v) by vigorous vortexing. A 90 μL sample was spun at 14,000 × *g* for 15 min at 4 °C, and the supernatant was injected into an ultra-high-performance liquid chromatography (UHPLC) system (Nexera LC-30A) interfaced with a Q Exactive mass spectrometer (Thermo Scientific^TM^) for comprehensive lipidomic analysis. All lipidomic components are presented in Supplementary Table 1.

#### Profiling of small-molecule constituents

Small-molecule profiling of CW-DNVs was performed by Novogene (Beijing, China). Metabolites were extracted from the nanoparticle samples and analyzed by liquid chromatography-mass spectrometry (LC-MS) on a high-sensitivity SCIEX QTRAP® 6500+ platform. Particular emphasis was placed on the identification and quantification of terpenoids, enabling a comprehensive survey of their classes and relative abundances. All small-molecule bioactive components are presented in Supplementary Table 2.

#### Thin-layer chromatography

CW-DNVs, curdione, curcumenol, atractylenolide II, and germacrone were each dissolved in dimethyl sulfoxide (DMSO) to a final concentration of 50 mg/mL. Aliquots of samples and standards were loaded onto silica gel plates (Qingdao Haiyang Chemical) and developed in a sealed chamber using a mobile phase consisting of petroleum ether and ethyl acetate (10:1, v/v). The run was stopped when the solvent front reached ~1 cm from the top (8-15 min). After drying, spots were visualized under the naked eye, then under UV light at 254 and 365 nm and photographed.

#### Small RNA sequencing

TRIzol reagent (Takara Bio, Dalian, China) was used to isolate total RNA from CW-DNVs. The RNA Nano 6000 Assay Kit (Agilent Technologies, USA) was employed to evaluate RNA quality and quantity. Subsequently, qualifying RNA samples were submitted to the Wuhan Huada Genomics Institute (Wuhan, China) for Small RNA (sRNA) library construction and sequencing on an Illumina platform. All sRNAs are presented in Supplementary Table 3.

#### Sodium dodecyl sulfate polyacrylamide gel electrophoresis profiling

Briefly, CW-DNVs proteins were extracted from the vesicles using Pierce IP lysis buffer (Thermo Scientific^TM^). Then, 40 μg of protein was resolved by Sodium dodecyl sulfate polyacrylamide gel electrophoresis (SDS-PAGE) on a 10% gel. After electrophoresis, the gel was stained with Coomassie Brilliant Blue solution and imaged using a gel imaging system.

#### Agarose gel electrophoresis of RNA

Equal amounts of CW-DNVs were divided into three aliquots. The first aliquot was left untreated, the second was treated with IP lysis buffer to facilitate release of internal contents, and the third was treated with IP lysis buffer followed by incubation with RNase A at 37 °C for 20 min to degrade RNA. All three samples were electrophoresed on a 1% agarose gel (100 V, 30 min), and the gel was subsequently imaged with a Bio-Rad ChemiDoc Imaging System.

### Cell culture and *in vitro* assays

#### Cell culture

AML12 and RAW264.7 cells were obtained from The Cell Bank of the Chinese Academy of Sciences, while mouse Kupffer cells were obtained from BeNa Culture Collection. Cells were cultured at 37 °C and 5% CO_2_ in specific media (DMEM/F12 for AML12, DMEM for RAW264.7, RPMI-1640 for Kupffer cells) supplemented with 10% fetal bovine serum and 1% penicillin-streptomycin.

#### Cell viability assay

The cytotoxicity of CW-DNVs was assessed using the 3-(4, 5-dimethylthiazol-2-yl)-2, 5-diphenyltetrazolium bromide (MTT) assay. RAW264.7 macrophages were seeded into 96-well plates at 1 × 10^4^ cells per well and cultured for 12 h. Cells were then treated with increasing concentrations of CW-DNVs (1.25, 2.5, 5, 10, 20 μg/mL) for 24 h, after which 20 μL of MTT solution (5 mg/mL) was added to each well and the plates were incubated at 37 °C for 4 h. The formazan crystals were dissolved in DMSO. Absorbance at 570 nm was measured using a microplate reader (SpectraMax iD3, Molecular Devices, USA), and cell viability was calculated as a percentage of control.

#### Hemolysis assay

After incubation of mouse erythrocytes with varying concentrations of CW-DNVs (5, 10, 20, and 40 μg/mL) for 4 h at 37 °C, the samples were centrifuged at 3,000 rpm for 15 min. Hemolysis was first judged visually; then the absorbance of the supernatant at 542 nm was recorded to calculate the percentage of hemolysis.

#### Cellular uptake of CW-DNVs

CW-DNVs were labeled with the red fluorescent dye PKH26 (Umibio, Shanghai, China) according to the manufacturer’s instructions. Briefly, a 1 mM PKH26 solution was diluted 10-fold with Diluent C under light-protected conditions to prepare a 100 μM working solution. Subsequently, 5 μL of this working solution was mixed with 200 μL of the vesicle suspension in PBS (1 μg/μL), and the resulting mixture was incubated for 30 min at room temperature in the dark for labeling. Subsequently, 38 mL of PBS was added, and the unbound PKH26 dye was removed by centrifugation at 15,000 × *g* for 90 min. Kupffer, RAW264.7, and AML12 cells were incubated with 10 μg/mL PKH26-labeled CW-DNVs for 12 or 24 h. After three washes with PBS, the cells were fixed with 4% paraformaldehyde and mounted in an antifade medium containing 4’,6-diamidino-2-phenylindole (DAPI) for nuclear staining. Cellular uptake was subsequently visualized by confocal microscopy (Nikon A1R, Japan) using a 60× oil-immersion lens.

#### sRNA and terpenoid function assays

RAW264.7 cells or Kupffer cells were seeded in 12-well plates and cultured for 12 h. Cells were further cultured for 24 h under the following conditions: (1) total sRNA isolated from CW-DNVs (transfected using Lipofectamine RNAiMAX); (2) individual terpenoids, including 50 μg/mL curdione, 50 μg/mL curcumenol, 50 μg/mL atractylenolide II, or 25 μg/mL germacrone; or (3) an equimolar mixture of these terpenoids. After pretreatment, cells were stimulated with lipopolysaccharide (LPS) to induce inflammation, and cytokine transcript levels were quantified by real-time quantitative PCR (RT-qPCR).

#### Lipid accumulation assay

AML12 cells were seeded in 12-well plates and cultured for 12 h. The cells were then incubated with CW-DNVs (10 μg/mL) for 24 h, followed by treatment with 200 μM oleic acid (OA) to induce lipid accumulation. Lipid droplet formation was quantified using Oil Red O (ORO) staining.

### Animal experiments and *in vivo* evaluation

#### Animal model and treatment

Male C57BL/6J mice, weighing 20-22 g, were used in this study, and all experimental procedures received approval from the Ethics Committee of Wenzhou Medical University (No. xmsq2024-0611). Mice were acclimatized for seven days prior to the experiment and then randomly assigned to four groups (*n* = 5), balanced by age and body weight. Two groups received a chow diet (normal control diet, NCD), while the other two were placed on a HFD (60% fat) for 15 weeks. Starting from week 8, one NCD group and one HFD group were injected intraperitoneally with 150 μg of CW-DNVs (NCD + CW-DNVs and HFD + CW-DNVs groups, respectively), administered once weekly for 7 consecutive weeks. The remaining two groups received equivalent volumes of PBS (NCD + PBS and HFD + PBS groups). All groups were observed for 15 weeks, during which body weight was recorded weekly. At the endpoint, mice were sacrificed using 2% sodium pentobarbital, and serum and liver tissues were collected for subsequent analysis.

#### Biodistribution of CW-DNVs

To evaluate the *in vivo* biodistribution of CW-DNVs, each mouse received a single intraperitoneal injection of 100 μg of Dil-labeled CW-DNVs. Whole-body fluorescence imaging was performed 12 h post-injection using a live-animal imaging system (IVIS Lumina XR, PerkinElmer, USA). At 12 h, 24 h, and 7 days post-treatment, mice were sacrificed, and liver, heart, lungs, kidneys, stomach, spleen, small intestine, and large intestine were dissected for imaging on a ChemiDoc MP system (Bio-Rad, USA).

#### Isolation of primary hepatic cells

For liver accumulation analysis, 100 μg of PKH26-labeled CW-DNVs were administered intraperitoneally. After 12 h, hepatocytes and Kupffer cells were isolated by a two-step collagenase perfusion method. The inferior vena cava was cannulated, and the liver was perfused sequentially with 5 mM EDTA buffer (37 °C), followed by 0.05% collagenase type IV (37 °C). The liver was removed, minced, and filtered through a 70 μm mesh. Hepatocytes were collected by centrifugation (50 × *g*, 3 min). Non-parenchymal cells were collected on a 50%/25% Percoll gradient, and Kupffer cells were harvested from the interface. The purity of the isolated Kupffer cells was verified to be > 90% by immunofluorescence using anti-CD68 (ab955, Abcam).

#### Biosafety evaluation

Mice received weekly intraperitoneal injections of 150 μg of CW-DNVs for seven weeks. Alanine aminotransferase (ALT), aspartate aminotransferase (AST), IL-6, and IL-1β levels were measured, and major organs were harvested for histopathological examination.

### Histological and biochemical analyses

#### Histological staining

Major organs were fixed overnight in 4% paraformaldehyde, embedded in paraffin, sectioned at a thickness of 5 μm, and stained with hematoxylin and eosin (HE) to assess tissue damage. The extent of histological change was evaluated as previously described^[30]^. ORO staining was performed to evaluate lipid accumulation. In short, after fixation in 10% formalin and propylene glycol treatment, frozen sections were stained with ORO (60 °C, 6 min), counterstained with modified Mayer’s hematoxylin, and imaged.

#### Immunohistochemical staining (IHC) and immunofluorescence

Paraffin-embedded liver tissue sections (4-5 µm) were baked and deparaffinized, and antigen retrieval was performed by pressure cooking in citrate buffer. Endogenous peroxidase activity was blocked with 3% H_2_O_2_, and nonspecific binding was blocked with 5% bovine serum albumin (BSA). The tissue sections were subsequently incubated overnight with primary antibodies targeting CD68 and IL-6. Immunoreactivity was detected using 3,3’-diaminobenzidine (DAB, followed by counterstaining with hematoxylin. Images were acquired using a Nikon microscope.

Mouse Kupffer cells were fixed overnight in 4% paraformaldehyde, followed by permeabilization with 0.5% Triton X-100 and blocking with 5% BSA. Subsequently, the cells were incubated with anti-CD68 antibody at a 1:200 dilution overnight at 4 °C, followed by incubation with Alexa Fluor 488-conjugated secondary antibody (1:500) for 1.5 h under dark conditions. Nuclear staining was performed with DAPI for 20 min. Images were acquired using a confocal microscope (Nikon A1R, Japan) equipped with a 60× oil-immersion objective.

#### Biochemical assays

Liver tissue supernatants, whole blood, and serum from mice were processed and analyzed in parallel. Commercial kits (Nanjing Jiancheng Bioengineering Institute) were used to measure serum levels of triglycerides (TG), total cholesterol (TC), ALT, AST, IL-6, and IL-1β in accordance with the manufacturer’s instructions. Fasting blood glucose (FBG) levels were assessed from tail-nick samples after 8-10 h fasting using Yuyue blood glucose test strips (Jiangsu Yuyue Medical Equipment & Supply Co., Ltd., China). For hepatic TG measurement, liver fragments were homogenized in ice-cold IP lysis buffer. The homogenate was kept on ice for 20 min, and then centrifuged at 12,000 × *g* for 20 min. The resulting supernatant was collected for TG quantification.

#### Western blot

For immunoblotting, total protein was extracted with IP/radioimmunoprecipitation assay (RIPA) buffer containing phenylmethylsulfonyl fluoride (PMSF) (Beyotime), and the protein concentration was measured by a bicinchoninic acid (BCA) assay (Beyotime). Following SDS-PAGE separation of equal protein amounts, the samples were transferred onto polyvinylidene fluoride (PVDF) membranes and blocked with 5% BSA. The membranes were then incubated overnight with primary antibodies against IL-6, peroxisome proliferator-activated receptor gamma coactivator 1-alpha (PGC-1α), microsomal triglyceride transfer protein (MTTP), fatty acid synthase (FASN), and glyceraldehyde 3-phosphate dehydrogenase (GAPDH), washed, and subsequently incubated with horseradish peroxidase (HRP)-conjugated secondary antibodies for 1.5 h. Blots were developed using enhanced chemiluminescence (ECL) and visualized with a ChemiDoc MP System (Bio-Rad).

#### RT-qPCR

Total RNA was extracted from cells or WYJ-DNs with TRIzol and 1 μg of RNA reverse-transcribed into cDNA using Vazyme HiScript III. RT-qPCR was carried out on a Bio-Rad CFX96 system with SYBR Master Mix (Vazyme). Relative mRNA expression levels were calculated using the 2^-ΔΔCt^ method with *Gapdh* as the internal control. The primers are shown in Supplementary Table 4.

### Statistical analysis

All data are presented as mean ± standard error of the mean (SEM). GraphPad Prism 10.0 was used for all statistical analyses. Differences between two groups were evaluated using an unpaired, two-tailed Student’s *t*-test, while comparisons involving three or more groups were performed using one-way analysisof variance (ANOVA) with Tukey’s post-hoc test. A significance threshold of *P* < 0.05 was adopted.

## RESULTS

### Isolation and characterization of CW-DNVs

**Figure 1:**
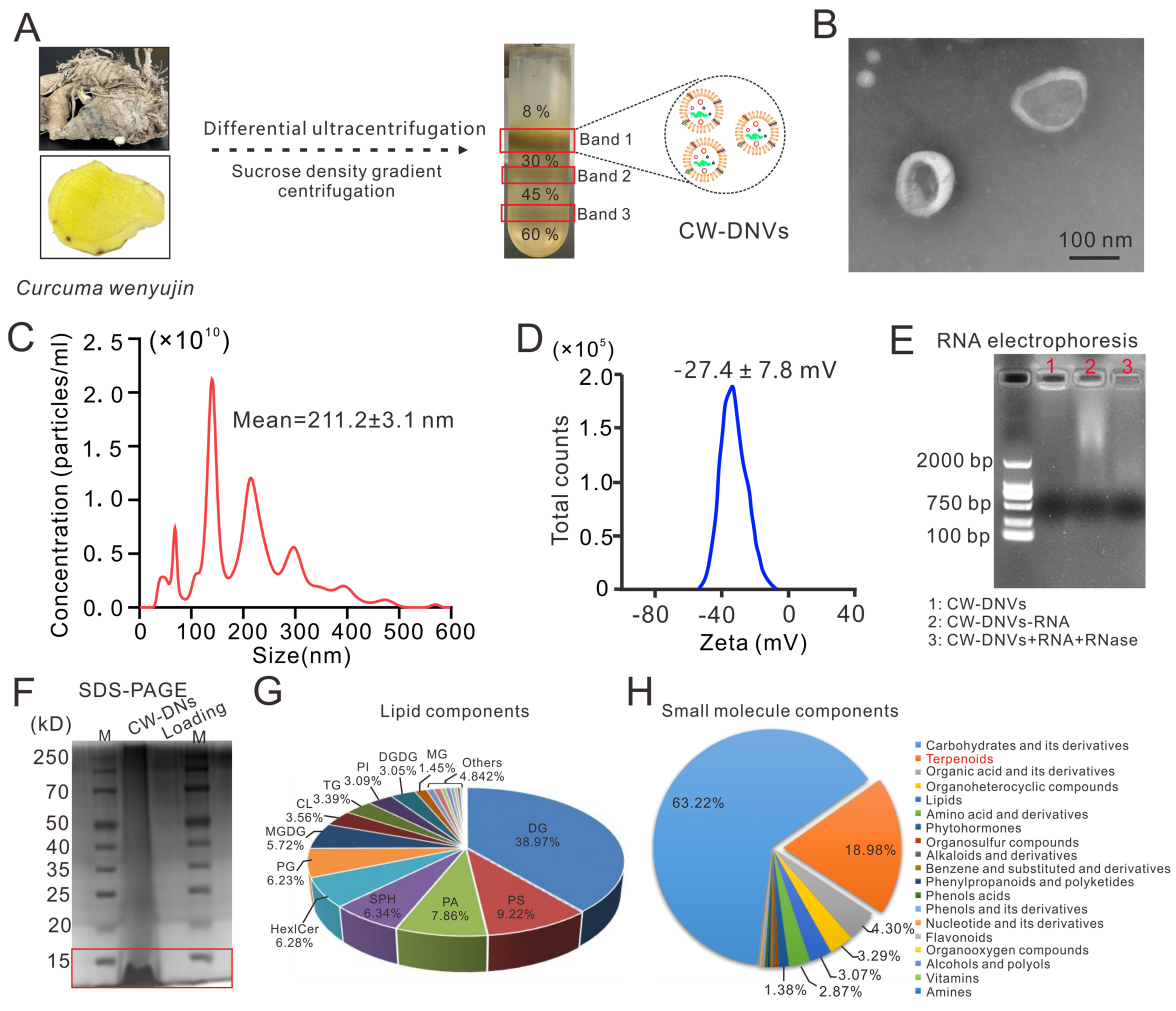
Characterization of CW-DNVs. (A) Schematic of CW-DNVs isolation from *C. wenyujin* rhizomes by differential ultracentrifugation and sucrose density gradient centrifugation. Band 1, hereafter referred to as CW-DNVs, was selected for further analysis; (B) Representative TEM image of CW-DNVs; (C) NTA showing size concentration and distribution and (D) zeta potential of CW-DNVs; (E) RNA presence assessed by agarose gel electrophoresis and (F) total proteins assessed by SDS-PAGE in CW-DNVs; (G) Lipidomics of CW-DNVs. Clockwise: DG, PS, PA, SPH, Hex1Cer, PG, MGDG, CL, TG, PI, DGDG, and MG; (H) Small-molecule constituents identified by LC-MS/MS with multiple reaction monitoring. CW-DNVs: *Curcuma wenyujin*-derived nanovesicles; TEM: transmission electron microscopy; NTA: nanoparticle tracking analysis; SDS-PAGE: sodium dodecyl sulfate-polyacrylamide gel electrophoresis; LC-MS/MS: liquid chromatography-tandem mass spectrometry; DG: diglycerides; PS: phosphatidylserine; PA: phosphatidic acid; SPH: sphingomyelin; Hex1Cer: hexosylceramide; PG: phosphatidylglycerol; MGDG: monogalactosyldiacylglycerol; CL: cardiolipin; TG: triglycerides; PI: phosphatidylinositol; DGDG: digalactosyldiacylglycerol; MG: monoglycerides.

CW-DNVs were successfully isolated from *C. wenyujin* through sequential ultracentrifugation under varying relative centrifugal forces and sucrose concentrations (8%, 30%, 45%, and 60% w/v). Following sucrose gradient ultracentrifugation, three distinct bands were observed, of which Band 1 (CW-DNVs-1) exhibited superior enrichment efficiency (0.242 mg/g) [Figure 1A]. CW-DNVs-1 were characterized by a significantly higher total vesicle mass compared to Band 2 (CW-DNVs-2) [Supplementary Figure 1A-C] and showed superior efficacy in inhibiting LPS-induced IL-6 production in macrophages [Supplementary Figure 1D], thus being selected for subsequent investigations. CW-DNVs-1 are hereafter referred to as CW-DNVs.

CW-DNVs exhibited a predominantly spherical morphology and were surrounded by a lipid bilayer [Figure 1B]. The CW-DNVs obtained from 50 g of fresh *C. wenyujin* rhizomes had an average diameter of 211.2 nm and reached a concentration of ~10^10^ particles/mL [Figure 1C and Supplementary Figure 1E]. Their zeta potential was determined to be -27.4 mV, indicative of moderate colloidal stability [Figure 1D]. The presence of RNA in CW-DNVs was confirmed via electrophoretic analysis [Figure 1E], while SDS-PAGE revealed that the protein profile was predominantly enriched in proteins below 15 kDa [Figure 1F]. The main phospholipid constituents were identified as diglycerides (DG, 38.97%), phosphatidylserine (PS, 9.22%), phosphatidic acid (PA, 7.86%), sphingomyelin (SPH, 6.32%), hexose ceramide (Hex1Cer, 6.28%), and phosphatidylglycerol (PG, 6.22%) through lipidomic profiling [Figure 1G]. Furthermore, MS analysis revealed that the major small-molecule components fell into the categories of carbohydrates and their derivatives, terpenoids, organic acids and their derivatives, organoheterocyclic compounds, and lipids [Figure 1H]. Collectively, these results demonstrate that nanoparticle-structured CW-DNVs with well-defined physicochemical properties and a complex molecular composition were successfully isolated from *C. wenyujin*.

### Accumulated delivery of CW-DNVs to Kupffer cells

**Figure 2:**
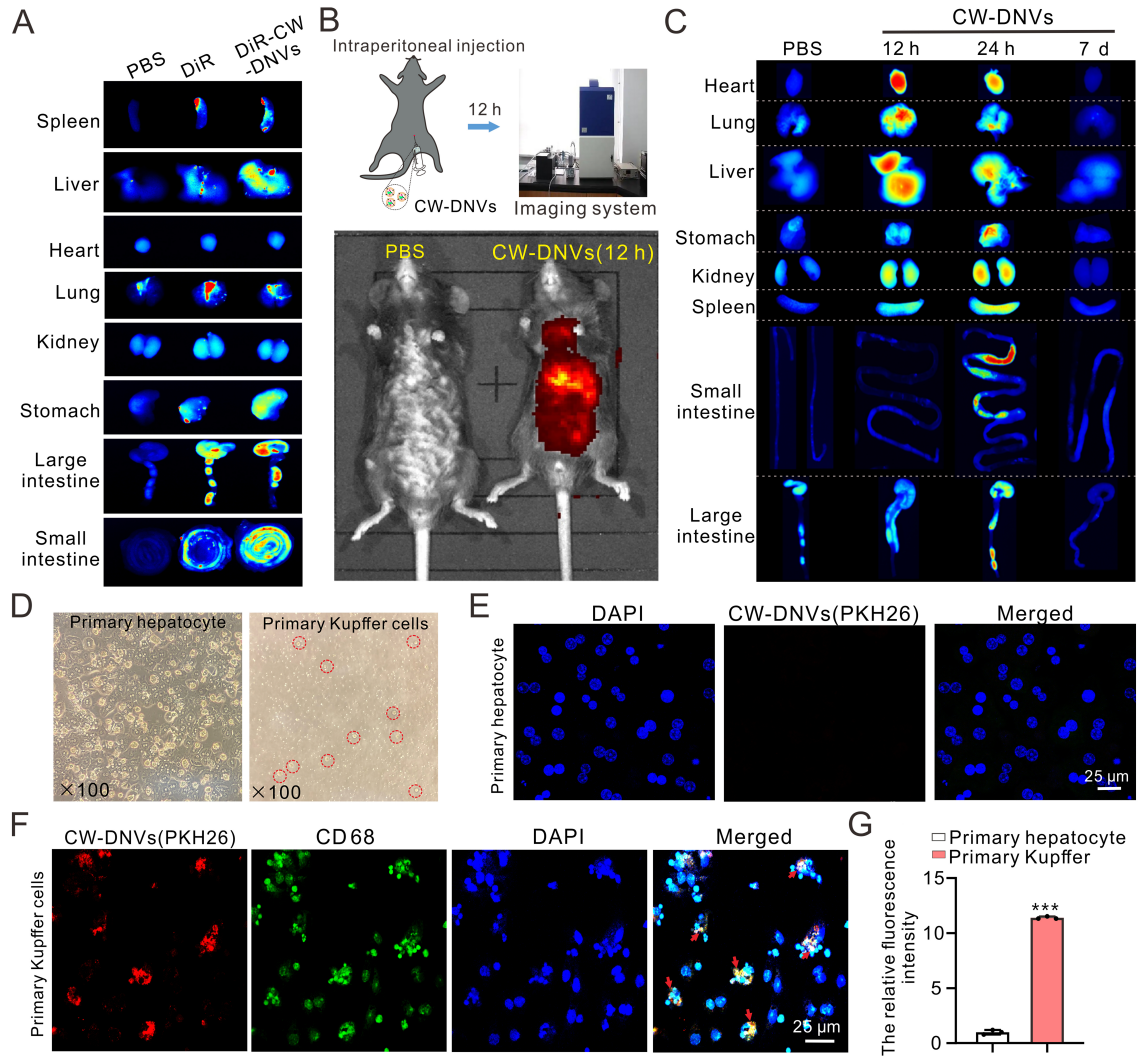
Accumulated delivery of CW-DNVs to liver macrophages. (A) DiR-labeled CW-DNVs were administered to mice via intraperitoneal injection, and biodistribution in organs was subsequently analyzed; (B) *In vivo* imaging at 12 h post-intraperitoneal injection of Dil-labeled CW-DNVs; (C) Biodistribution in organs 12 h, 24 h, and 7 day post-injection; (D) Isolated primary hepatocytes and KCs from mouse liver; (E) Uptake of PKH26-labeled CW-DNVs (red) by hepatocytes and (F) CD68+ KCs (green) 12 h post-treatment; (G) Relative fluorescence intensity of PKH26-labeled CW-DNVs in primary hepatocytes *vs*. KCs. Data represent the mean ± SEM (*n* = 3). Student’s *t*-test. ^***^P < 0.001. CW-DNVs: *Curcuma wenyujin*-derived nanovesicles; KCs: Kupffer cells; SEM: standard error of the mean; PBS: phosphate-buffered saline.

To assess the overall tissue distribution of CW-DNVs in mice, DiR-labeled CW-DNVs were administered via intraperitoneal injection, with free DiR dye serving as a control. The results showed significant enrichment of CW-DNVs in the liver [Figure 2A]. Subsequently, Dil-labeled CW-DNVs (100 μg per mouse) were administered via intraperitoneal injection to examine regional accumulation. At 12 h after injection, *in vivo* imaging revealed a predominant accumulation of CW-DNVs in the abdominal region [Figure 2B]. At the same time point, CW-DNVs localized primarily in the liver, lungs, and heart, with lower fluorescence levels in the kidneys, spleen, and stomach. At 24 h after injection, the fluorescence signal had diminished in the liver, heart, and lungs, whereas it had increased in the kidneys, stomach, and spleen; minor fluorescence accumulation was also detected in intestinal tissues. This shift in fluorescence signal is suggestive of ongoing systemic clearance of CW-DNVs. By day 7 post-injection, no fluorescence was detected in any harvested organ, indicating complete elimination of CW-DNVs (Figure 2C; quantitative data for each organ are provided in Supplementary Figure 2A).

Given the robust hepatic accumulation observed shortly after injection, primary hepatocytes (predominantly multinucleated) and Kupffer cells (mononuclear, indicated by red circles in Figure 2D) were subsequently isolated. Following incubation with PKH26-labeled CW-DNVs, negligible fluorescence was detected in hepatocytes [Figure 2E], whereas strong PKH26 signal with distinct co-localization was observed in CD68-positive Kupffer cells [Figure 2F, Supplementary Figure 2B and C]. Quantitative analysis confirmed that the uptake efficiency of CW-DNVs in Kupffer cells was 11.40-fold higher than that in hepatocytes [Figure 2G]. These results demonstrate that CW-DNVs administered via intraperitoneal injection preferentially localize to hepatic macrophages in mice.

### CW-DNVs alleviate HFD-induced hepatic steatosis in mice

**Figure 3:**
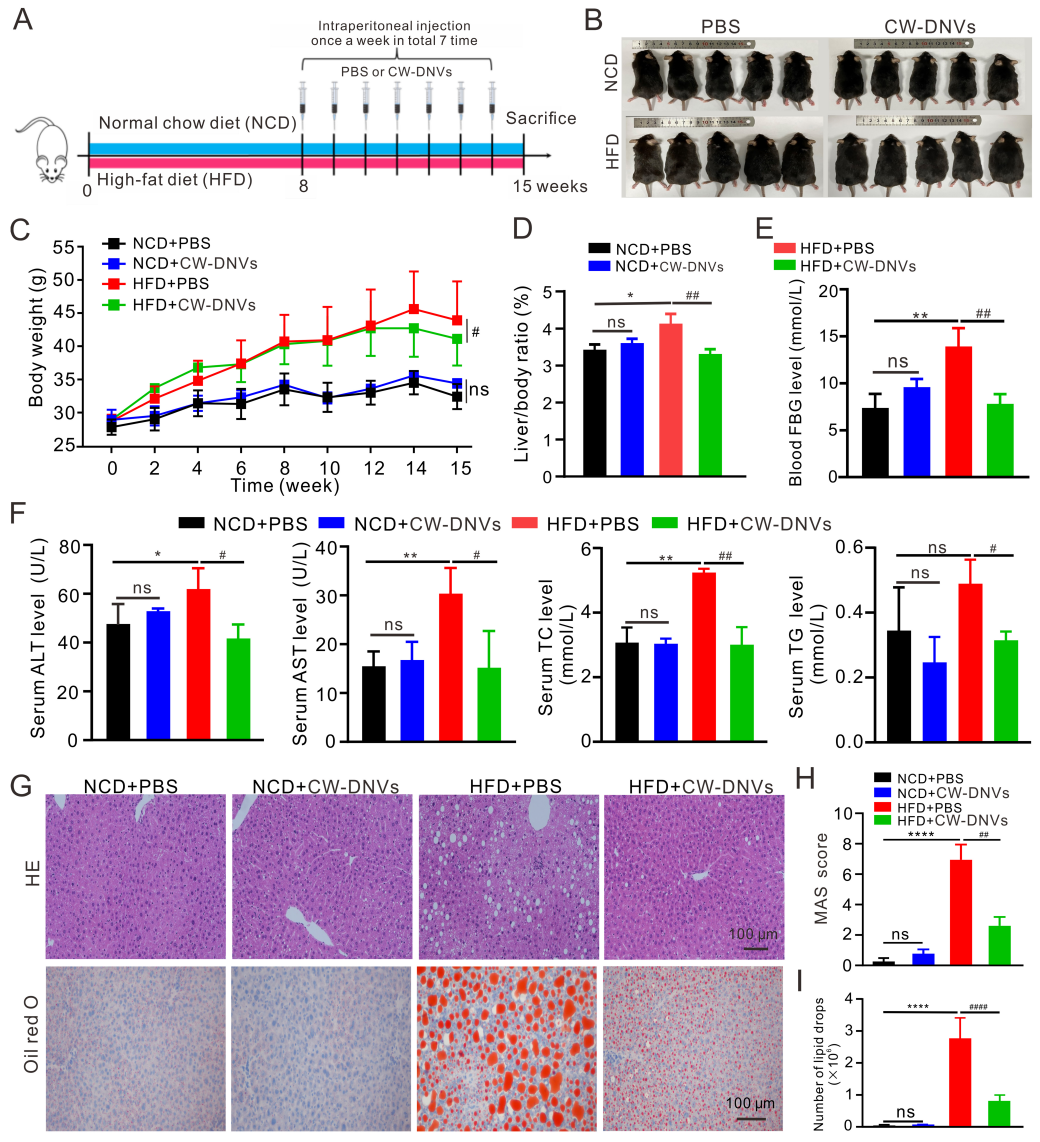
CW-DNVs ameliorate HFD-induced MASH. (A) Experimental design; (B) Body gross appearance; (C) Body weight changes; (D) Liver-to-body weight ratio; (E) Fasting blood glucose levels; (F) Serum levels of AST, ALT, TG, and TC; (G) Representative images of HE- and ORO-stained liver sections; (H) MAS; (I) Quantitative analysis of the ORO-positive lipid droplet area. Values represent the mean ± SEM (*n* = 5). One-way ANOVA with Tukey’s multiple-comparison test. ^*^P < 0.05; ^**^P < 0.01; ^***^P < 0.001; ^****^P < 0.0001 *vs*. NCD + PBS group; ^#^*P* < 0.05; ^##^*P* < 0.01; ^####^*P* < 0.0001 *vs*. HFD + PBS group. CW-DNVs: *Curcuma wenyujin*-derived nanovesicles; MASH: metabolic dysfunction-associated steatohepatitis; AST: aspartate aminotransferase; ALT: alanine aminotransferase; TG: triglycerides; TC: total cholesterol; HE: hematoxylin and eosin; ORO: Oil Red O; MAS: MAFLD Activity Score; MAFLD: metabolic dysfunction-associated fatty liver disease; SEM: standard error of the mean; ANOVA: analysis of variance; NCD: normal chow diet; PBS: phosphate-buffered saline; HFD: high fatty diet; ns: not significant.

Having established that CW-DNVs preferentially accumulate in hepatic macrophages, their therapeutic effects on MASH were investigated in an HFD-induced mouse model. Mice were divided into four groups: NCD + PBS, NCD + CW-DNVs (150 μg), HFD + PBS, and HFD + CW-DNVs (150 μg). As outlined in Figure 3A, after 8 weeks of HFD (60% fat) feeding, mice were administered weekly intraperitoneal injections of 150 μg of CW-DNVs for 7 weeks. All mice were sacrificed at week 15, and serum biochemical analyses and histopathological examinations were performed. Treatment with CW-DNVs attenuated the HFD-induced increase in body weight and weight gain [Figure 3B and C], and ameliorated the steatotic liver phenotype as well as the elevated liver-to-body weight ratio [Figure 3D and Supplementary Figure 3]. Mice fed an HFD exhibited elevated fasting glucose levels (13.6 ± 1.8 mmol/L), which were reduced to 8.1 ± 0.8 mmol/L following CW-DNVs treatment [Figure 3E]. In addition, CW-DNVs significantly reversed HFD-induced elevations in serum ALT, AST, TG, and TC levels [Figure 3F], indicating improved hepatic function and lipid metabolic homeostasis. Histological analyses revealed that HFD feeding led to extensive hepatic lipid vacuolation and inflammatory cell infiltration, while ORO staining revealed pronounced intrahepatic accumulation of lipid droplets. These histopathological alterations were largely reversed by CW-DNVs treatment [Figure 3G]. Quantitative analysis showed that administration of CW-DNVs to HFD-fed mice reduced the MAFLD Activity Score from 7.2 to 2.8 [Figure 3H] and decreased the intrahepatic lipid droplet count from 2.8 × 10^6^ to 0.86 × 10^6^ [Figure 3I]. Collectively, these findings demonstrate that CW-DNVs effectively attenuate HFD-induced hepatic steatosis.

### CW-DNVs attenuate hepatic steatosis via dual modulation of lipid metabolism and inflammation

**Figure 4:**
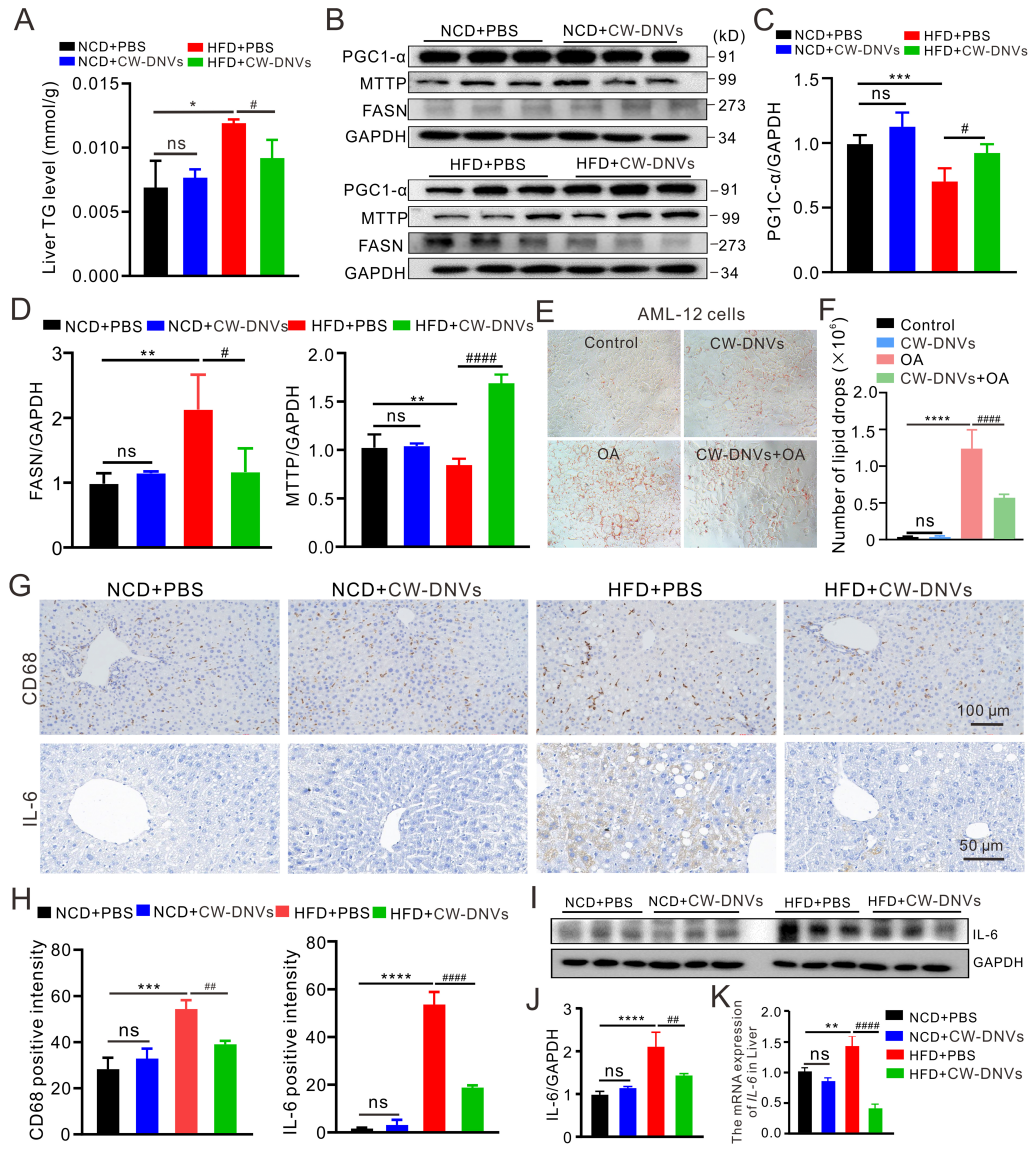
Molecular mechanisms of CW-DNVs in MASH attenuation. (A) Hepatic TG content; (B-D) Western blot and quantification of PGC-1α, FASN, and MTTP in liver; (E and F) Lipid droplet formation in AML12 cells treated with CW-DNVs and OA; (G and H) Immunohistochemistry for CD68 and IL-6 in liver sections; (I-K) Hepatic IL-6 expression assessed by Western blot (I and J) and qPCR (K). Data are presented as mean ± SEM (*n* = 3 for *in vitro* studies; *n* = 5 mice for *in vivo* studies). One-way ANOVA with Tukey’s multiple-comparison test. ^*^P < 0.05; ^**^P < 0.01; ^***^P < 0.001; ^****^P < 0.0001 *vs.* NCD + PBS or Control group; ^#^*P* < 0.05; ^##^*P* < 0.01; ^####^*P* < 0.0001 *vs.* HFD + PBS or OA alone group. CW-DNVs: *Curcuma wenyujin*-derived nanovesicles; MASH: metabolic dysfunction-associated steatohepatitis; TG: triglycerides; PGC-1α: peroxisome proliferator-activated receptor gamma coactivator 1-alpha; FASN: fatty acid synthase; MTTP: microsomal triglyceride transfer protein; AML12: alpha mouse liver 12; OA: oleic acid; IL-6: interleukin-6; qPCR: quantitative polymerase chain reaction; SEM: standard error of the mean; ANOVA: analysis of variance; NCD: normal chow diet; PBS: phosphate-buffered saline; CD68: cluster of differentiation 68; HFD: high fatty diet; ns: not significant.

To investigate the molecular mechanisms underlying the anti-steatotic effect of CW-DNVs, hepatic TG content was quantified. Feeding an HFD increased the hepatic TG content to 0.012 mmol/g, which was reduced to 0.009 mmol/g upon treatment with CW-DNVs [Figure 4A]. Western blot analysis of lipid metabolism-related proteins showed that HFD downregulated PGC-1α and MTTP, whereas the lipogenic enzyme FASN was upregulated. These alterations were reversed by treatment with CW-DNVs, which shifted the balance toward promoting β-oxidation, enhancing very low-density lipoprotein (VLDL) transport, and diminishing *de novo* lipogenesis [Figure 4B-D]. In AML12 hepatocytes, where CW-DNVs were effectively internalized [Supplementary Figure 4], lipid droplet accumulation induced by OA was alleviated by pre-treatment with CW-DNVs [Figure 4E and F], supporting a direct effect on the regulation of hepatocyte lipid homeostasis. HFD-induced hepatic inflammation was also attenuated by CW-DNVs treatment. Immunostaining for CD68 and IL-6 showed that the proportion of CD68-positive macrophages decreased from 57.4% to 40.8%, while IL-6-positive cells were reduced from 58.2% to 19.6% following CW-DNVs treatment [Figure 4G and H]. Consistently, the HFD-induced upregulation of hepatic IL-6 mRNA and protein was markedly attenuated by CW-DNVs [Figure 4I-K]. Taken together, these results demonstrate that CW-DNVs mitigate HFD-induced hepatic steatosis through a dual mechanism involving regulation of lipid metabolism and suppression of inflammation.

### The anti-inflammatory effect of CW-DNVs can be attributed primarily to their cargo of small molecular weight terpenoids

**Figure 5:**
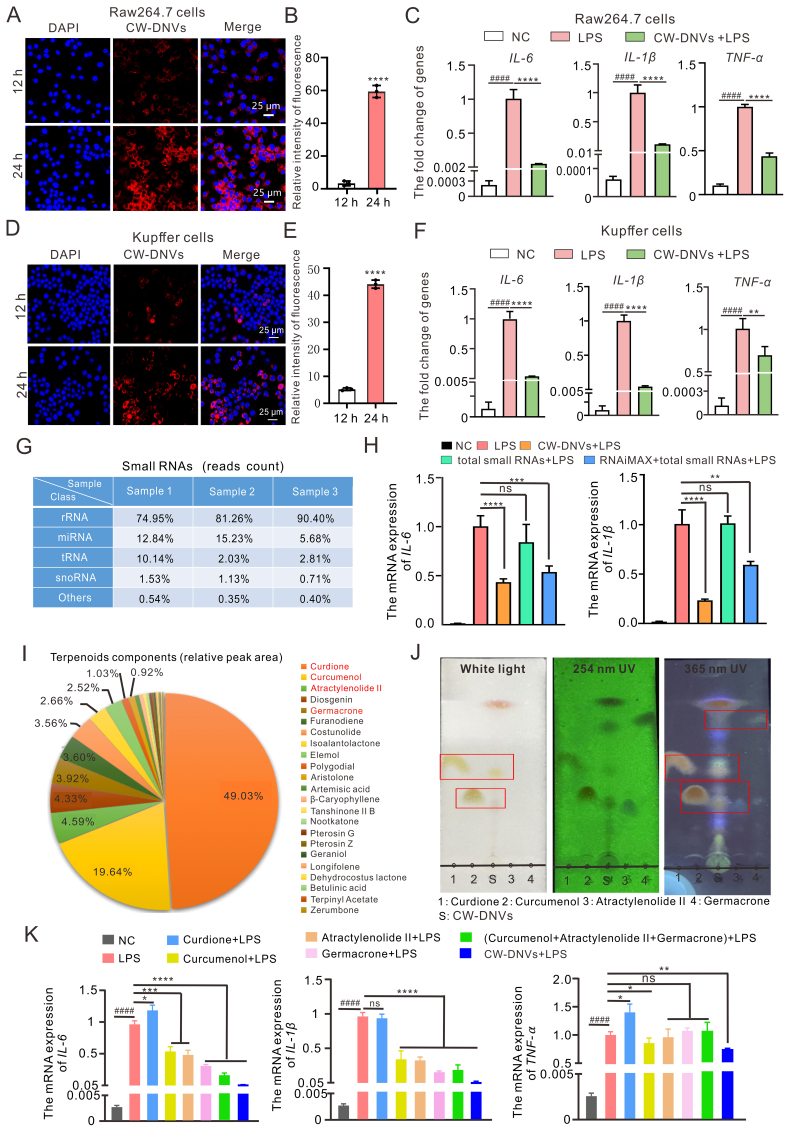
CW-DNVs alleviate inflammation through sRNA cargo and terpenoid components. (A) Confocal images and (B) quantification of Raw264.7 cells after incubation with PKH26-labeled CW-DNVs; (C) Expression of Il6, Il1b, and Tnfα mRNA in Raw264.7 cells following pretreatment with CW-DNVs and subsequent LPS stimulation; (D) Confocal images and (E) quantification of primary KCs incubated with PKH26-labeled CW-DNVs; (F) Expression of Il6, Il1b, and Tnfα mRNA in KCs following CW-DNVs pretreatment and LPS stimulation; (G) RNA sequencing read counts for sRNA classes in CW-DNVs; (H) Il6 and Il1b mRNA quantification in KCs transfected with total sRNA isolated from CW-DNVs, followed by LPS stimulation; (I) LC-MS peak-area quantification of terpenoids in CW-DNVs; (J) TLC identification of curdione, curcumenol, atractylenolide II, and germacrone; (K) Effects of individual terpenoids or an equimolar mixture on the expression of LPS-induced inflammatory cytokine genes (Il6, Il1b, and Tnfα) in macrophages. Data are presented as mean ± SEM (*n* = 3). One-way ANOVA with Tukey’s multiple-comparison test. ^####^*P* < 0.0001 *vs.* NC group. ^*^P < 0.05; ^**^P < 0.01; ^***^P < 0.001; ^****^P < 0.0001 *vs.* LPS-only group. CW-DNVs: *Curcuma wenyujin*-derived nanovesicles; sRNA: small RNA; KCs: Kupffer cells; LPS: lipopolysaccharide; LC-MS: liquid chromatography-mass spectrometry; TLC: thin-layer chromatography; SEM: standard error of the mean; ANOVA: analysis of variance; NC: negative control; ns: not significant.

To define the anti-inflammatory capacity of CW-DNVs in macrophages and elucidate the contribution of their core components, their efficacy was first established in Raw264.7 cells. A concentration-dependent suppression of inflammatory cytokines was observed after 24 h pretreatment with CW-DNVs and subsequent LPS challenge. Significant downregulation of *Il1b* was noted at concentrations ≥ 3 μg/mL, with maximal anti-inflammatory effects achieved at 10 μg/mL [Supplementary Figure 5]. This optimal concentration was therefore adopted for all subsequent studies. Efficient intracellular uptake by Raw264.7 cells was confirmed by time-course imaging of PKH26-labeled CW-DNVs between 12 h and 24 h [Figure 5A and B]. Consistently, potent suppression of LPS-induced transcription of *Il6*, *Il1b*, and *Tnf-α* (encoding IL-6, IL-1β, and TNF-α, respectively) was observed after a 24-h pretreatment with CW-DNVs (10 μg/mL) [Figure 5C]. An identical uptake pattern and comparable cytokine suppression were observed in Kupffer cells [Figure 5D-F], confirming conserved anti-inflammatory effects of CW-DNVs across macrophage types.

The anti-inflammatory activity of PDNVs is often attributed primarily to their miRNA cargo^[31,32]^. In the case of CW-DNVs, sRNA sequencing revealed a complex RNA landscape, with rRNA-derived sRNAs constituting the majority [Figure 5G]. Additionally, a modest suppressive effect on *Il6* and *Il1b* expression was observed after transfection of the isolated total sRNA into Kupffer cells [Figure 5H]. However, the available evidence indicates that the terpenoid components are the primary mediators of the potent anti-inflammatory effects. This conclusion is strongly supported by subsequent compositional and functional analyses. A characteristic terpenoid profile was revealed by LC-MS analysis, with curdione being the most abundant metabolite (49.03%), followed by curcumenol (19.64%), atractylenolide II (4.59%), diosgenin (4.33%), and germacrone (3.92%) [Figure 5I]. These components were further verified by thin-layer chromatography (TLC), which showed intense signals for curdione, curcumenol, and germacrone, consistent with the LC-MS data [Figure 5J]. Functional validation in mouse Kupffer cells demonstrated that LPS-stimulated *Il6* and *Il1b* expression was markedly reduced by pretreatment with curcumenol, atractylenolide II, or germacrone, either individually or as an equimolar mixture. Significant suppression of *Tnf* was observed with the equimolar mixture and with curcumenol alone [Figure 5K], underscoring the critical role of these terpenoids. Collectively, these findings demonstrate that CW-DNVs function as a natural repository predominantly enriched in terpenoids, highlighting their potential as a novel and efficient delivery vehicle and combinatorial therapeutic approach for mitigating inflammation.

### Biocompatibility and biosafety evaluation of CW-DNVs

To assess the *in vitro* biocompatibility of CW-DNVs, the viability of Raw264.7 cells was assessed by MTT assay following incubation with different concentrations of the nanovesicles. The results demonstrated no significant cytotoxicity across the range of concentrations tested [Supplementary Figure 6A]. Hemocompatibility was further examined via a standard hemolysis assay. While distilled water induced severe hemolysis (positive control), CW-DNVs at all concentrations induced hemolysis levels comparable to the PBS negative control [Supplementary Figure 6B], confirming excellent blood compatibility. For *in vivo* systemic safety evaluation, mice received weekly intraperitoneal injections of CW-DNVs for 1 month. Subsequent serum biochemical analysis revealed no significant changes in key markers of liver function (AST and ALT; Supplementary Figure 6C) or systemic inflammation (*Il6* and *Il1b* expression; Supplementary Figure 6D). Furthermore, thorough histopathological evaluation did not reveal any structural abnormalities or tissue damage in the major organs (including liver, heart, lung, spleen, and kidney) of CW-DNVs-treated animals [Supplementary Figure 6E]. Collectively, these results demonstrate that CW-DNVs exhibit negligible cytotoxicity, hemolytic activity, and organ toxicity, thereby supporting their favorable biosafety profile for potential therapeutic applications.

## DISCUSSION

This study demonstrates that CW-DNVs alleviate MASH by specifically accumulating in hepatic macrophages and suppressing inflammation through multiple mechanisms. Following intraperitoneal administration, CW-DNVs predominantly accumulate in the liver, exhibiting a distinct tropism for hepatic macrophages (Kupffer cells). This preferential accumulation of CW-DNVs is driven by a combination of their unique physicochemical properties and the liver’s physiological clearance mechanisms. Specifically, (1) their size (~211 nm) is well-suited for passive capture in the liver sinusoids; (2) their negative surface charge (-27.4 mV) likely promotes opsonization and recognition by scavenger receptors on Kupffer cells; and (3) their membrane composition, particularly PS, which acts as an “eat-me” signal, facilitates specific phagocytosis^[33,34]^. Collectively, these attributes enable efficient accumulation and clearance of CW-DNVs by the liver’s reticuloendothelial system. Such targeted delivery not only enhances therapeutic efficacy but also significantly minimizes off-target risks, representing a major advantage of this nanotherapeutic strategy. Future research should elucidate the precise molecular recognition pathways that govern this natural tropism to optimize delivery efficiency for clinical translation.

The anti-inflammatory efficacy of CW-DNVs is evidenced by marked reduction of IL-6, IL-1β, and TNF-α in both HFD-induced MASH mouse models and LPS-stimulated macrophages. Our compositional analysis indicates that this potent activity stems from a synergistic interplay among various bioactive cargoes. Notably, CW-DNVs encapsulate a natural and diverse combination of terpenoids, effectively concentrating the functional small molecules of *C. wenyujin* into a bioavailable nanoplatform. Specifically, key terpenoids enriched within the vesicles, such as curcumenol, atractylenolide II, and germacrone, significantly inhibited the expression of IL-6 and IL-1β in Kupffer cells. A total of 169 compounds, ranging from mono-, sesqui-, and diterpenoids to curcuminoids and others, have been isolated from *C wenyujin* thus far^[29]^. In parallel, findings from pharmacological investigations and clinical studies have revealed that *C. wenyujin* extracts exert anti-inflammatory, antioxidant, and hepatoprotective effects^[29]^. Among the identified compounds, curcumenol and curcumolide showed anti-inflammatory effects, while curdione and germacrone demonstrated hepatoprotective properties^[28,35-37]^. Our findings on the anti-inflammatory effects of curcumenol, atractylenolide II, and germacrone are consistent with these studies and further extend the understanding of the bioactive terpenoids in *C. wenyujin*. Furthermore, while sRNA sequencing identified a complex RNA profile dominated by rRNA-derived fragments, the collective anti-inflammatory effects observed in the present study strongly suggest that these rRNA-derived sRNAs possess critical biological functions rather than representing mere degradation byproducts^[38-40]^. This multi-component, synergistic mechanism, integrating specific terpenoid assemblies with functional sRNAs, exemplifies a novel therapeutic approach that may offer a more robust intervention than conventional single-target therapies.

In contrast to previous reports that emphasize miRNAs as key mediators in other PDNVs, our study reveals that the anti-inflammatory activity of CW-DNVs can be primarily attributed to small-molecule terpenoids, for example, germacrone and curcumenol, rather than to miRNAs. This discrepancy likely reflects species-specific metabolic profiles, differences in isolation and analytical methods, and potential synergistic effects among vesicle components. Our findings highlight the compositional diversity of PDNVs and underscore the importance of secondary metabolites as key bioactive agents in CW-DNVs for anti-inflammatory therapy.

Overall, the proposed synergistic mechanism provides a theoretical and practical framework for treating MASH. Comprehensive biosafety assessments confirm that CW-DNVs exhibit excellent biocompatibility, with no detectable systemic toxicity, supporting their potential as a naturally derived nanomedicine. Notably, the therapeutic landscape for MASH has evolved rapidly. Resmetirom (Rezdiffra) received accelerated FDA approval in March 2024 for adults with noncirrhotic MASH and moderate-to-advanced fibrosis (F2-F3)^[5]^. In August 2025, the FDA also approved semaglutide (Wegovy) for MASH with moderate-to-advanced liver fibrosis, further expanding treatment options^[6-8]^. While these regulatory breakthroughs provide essential pharmaceutical options, the field still requires diverse and well-tolerated modalities. CW-DNVs represent a promising alternative or complementary strategy within the expanding MASH therapeutic arsenal. Nevertheless, it should be acknowledged that direct head-to-head comparisons between CW-DNVs and conventional treatments (such as resmetirom or semaglutide) were not within the scope of the present study. Future investigations should systematically compare the efficacy, safety, and cost-effectiveness of CW-DNVs with these established therapies to determine whether CW-DNVs offer superior or equivalent therapeutic benefits. Key future challenges include verifying long-term safety in human trials and fully mapping the molecular pathways of all bioactive components.

Several limitations should be acknowledged. First, the synergistic contributions of individual terpenoids and sRNAs to the overall anti-inflammatory effect of CW-DNVs were not quantitatively dissected. Second, the molecular mechanisms underlying the preferential targeting of Kupffer cells remain unclear. Third, long-term safety and potential immunogenicity of CW-DNVs were only evaluated over four weeks. Fourth, the ultracentrifugation-based isolation method limits scalability for clinical production. Addressing these issues will be critical for future translation.

In summary, CW-DNVs represent a promising naturally derived nanomedicine for MASH. They offer superior biocompatibility and low toxicity compared to synthetic alternatives, while their multicomponent composition enables synergistic, multitargeted anti-inflammatory effects. With cost-effective production and inherent liver-targeting capability, CW-DNVs help overcome the bioavailability limitations of lipophilic phytochemicals. Beyond MASH, their immunomodulatory properties hold potential for diverse inflammatory conditions. Future optimization and clinical validation will be essential to advance CW-DNVs as an efficient and sustainable therapeutic option.

Future research should focus on three key areas: First, optimizing the isolation of active components from CW-DNVs and integrating multi-omics approaches to elucidate their synergistic mechanisms. Second, employing gene-editing and proteomic tools to identify the specific molecular targets through which CW-DNVs regulate inflammatory and antioxidant signaling pathways. Third, accelerating preclinical studies in large-animal models and advancing clinical trials to expand their application in inflammatory diseases, alongside long-term safety assessments to support clinical translation.

## DECLARATIONS

### Availability of data and materials

The raw data supporting the findings of this study are available within this Article and its Supplementary Materials. Further data are available from the corresponding authors upon request.

### AI and AI-assisted tools statement

During the preparation of this manuscript, the AI tool DeepSeek (version 4, released 2026-07-24) was used solely for language editing. The tool did not influence the study design, data collection, analysis, interpretation, or the scientific content of the work. All authors take full responsibility for the accuracy, integrity, and final content of the manuscript.

### Copyright

© The Author(s) 2026.
