# Supplementary material for: Terpenoid-enriched Curcuma wenyujin nanovesicles for suppressing inflammation and restoring lipid homeostasis in MASH
Source: Extracell Vesicles Circ Nucl Acids. 2026 Jun 30;7(2):1010–28. doi: 10.20517/evcna.2025.181 (PMC13367161; doi:10.20517/evcna.2025.181)
Supplement: Supplementary file 1 [file evcna-7-2-1010-SupplementaryMaterials.zip › evcna60181-SupplementaryMaterials/evcna60181-SupplementaryMaterials.pdf]

Supplementary Materials

Terpenoid-enriched *Curcuma wenyujin* nanovesicles for suppressing inflammation and restoring lipid homeostasis in MASH

Lin Liu[1,#], Jiale Niu[1,#], Yulong Sun[1], Zhuoyan He[1], Ziming Jiao[1], Guoen Li[1], Ganglin Wang[1], Fangjun Luo[2], Wei Li[1]

[1]Key Laboratory of Laboratory Medicine, Ministry of Education of China, Zhejiang Provincial Key Laboratory of Medical Genetics, School of Laboratory Medicine and Life Sciences, Wenzhou Medical University, Wenzhou 325035, Zhejiang, China.
[2]Department of Clinical Laboratory, Zhuji People's Hospital of Zhejiang Province, Zhuji 311800, Zhejiang, China.
[#]These authors contributed equally to this work.

Correspondence to: Dr. Fangjun Luo, Department of Clinical Laboratory, Zhuji People's Hospital of Zhejiang Province, Zhuji 311800, Zhejiang, China. E-mail: zjzjlfj@163.com; Dr. Wei Li, Key Laboratory of Laboratory Medicine, Ministry of Education of China, Zhejiang Provincial Key Laboratory of Medical Genetics, School of Laboratory Medicine and Life Sciences, Wenzhou Medical University, Wenzhou 325035, Zhejiang, China. E-mail: wei.li@wmu.edu.cn

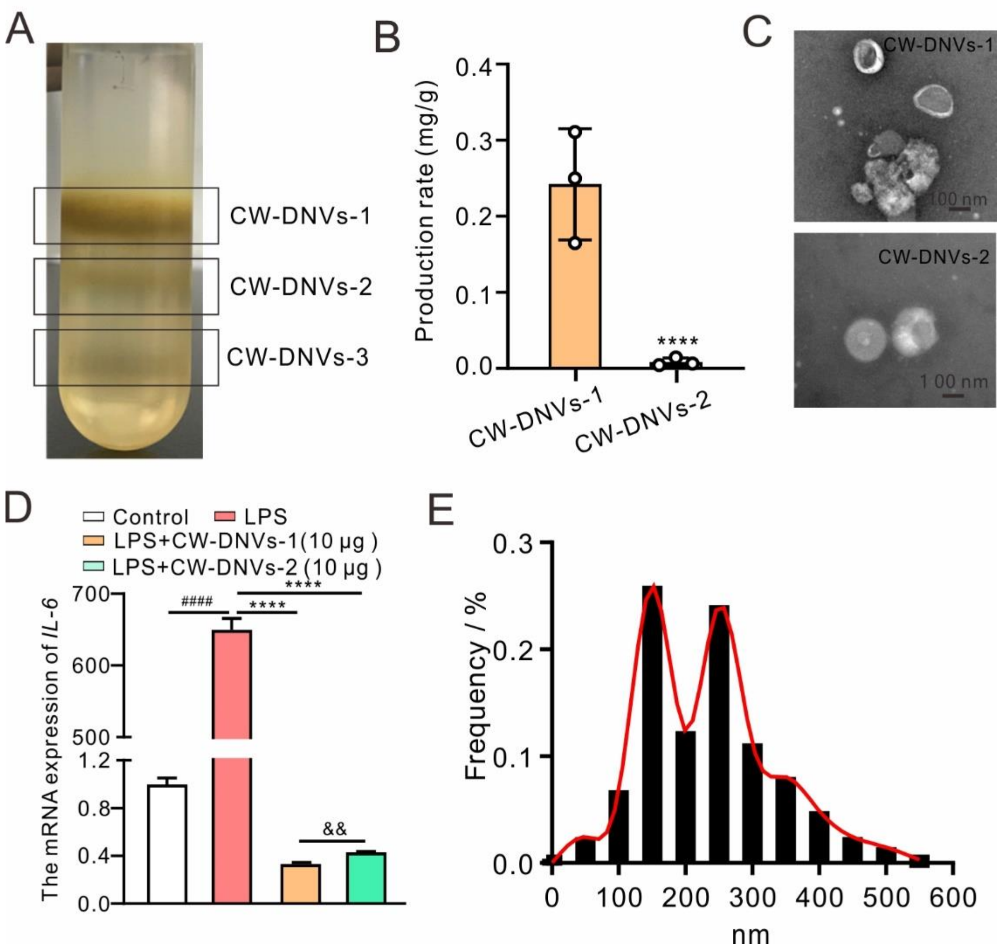

Supplementary Figure 1. CW-DNVs yield and anti-inflammatory potency. (A) Sucrose-density-gradient profile showing the three CW-DNVs bands; (B) CW-DNVs - 1 yielded 34-fold more nanoparticles than CW-DNVs-2. $^{****}P < 0.0001$ *vs.* CW-DNVs-1; (C) TEM image of CW-DNVs -1 and CW-DNVs-2; (D) At equal protein dose (10 μg), CW-DNVs-1 suppressed LPS-induced IL-6 more strongly than CW-DNVs-2. Data are presented as mean ± SEM (n = 3). Statistical significance was assessed by an unpaired, two-tailed Student's *t*-test or One-way ANOVA with Tukey's multiple-comparison test. $^{\#\#\#\#}P < 0.0001$ *vs.* control, $^{****}P < 0.0001$ *vs.* LPS alone; $^{\&\&}P < 0.01$ *vs.* CW-DNVs-1.

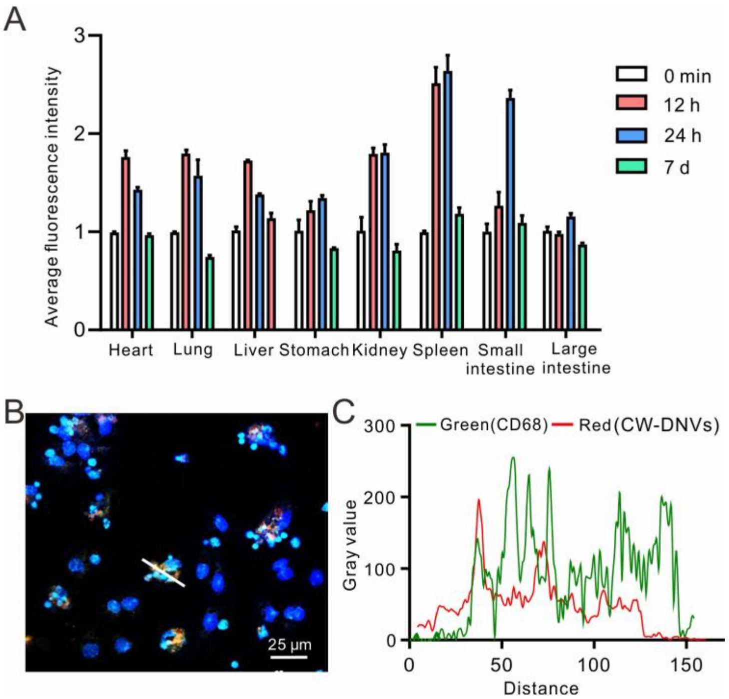

Supplementary Figure 2. Following intraperitoneal administration, CW-DNVs biodistribution across organs was tracked at serial time points (A), Laser-scanning confocal microscopy then visualized their co-localization with $CD68^+$ Kupffer cells (B) and enabled quantitative analysis (C). Data are presented as mean ± SEM (n = 3).

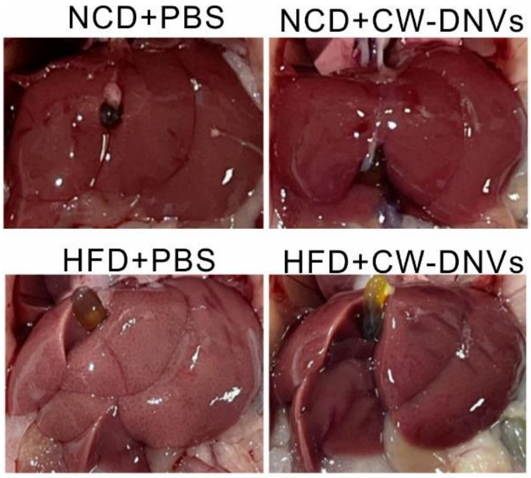

Supplementary Figure 3. Representative liver photographs from the four experimental groups: NCD + PBS, NCD + CW-DNVs, HFD + PBS, and HFD + CW-DNVs.

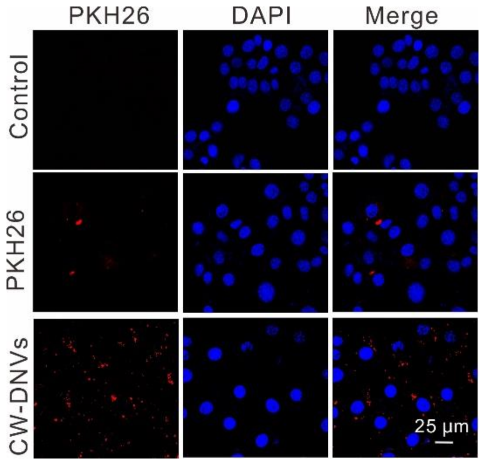

Supplementary Figure 4. Fluorescence micrograph of AML12 cells following 24 h co-incubation with PKH26-labeled CW-DNVs (10 μg/mL) or PKH26 dye. For free PKH26 staining, 1 μL of the 100 μM PKH26 working solution was co-incubated with AML12 cells. For vesicle labeling, 1 μL of the same PKH26 working solution (100 μM) was mixed with 20 μL of CW-DNVs (10 μg/mL) and incubated in the dark for 30 minutes. Unbound PKH26 dye was then removed by centrifugation at 15,000 × *g* for 90 min. Finally, both free PKH26 dye and PKH26-labeled CW-DNVs were separately co-incubated with AML12 cells for 24 h and then imaged using confocal laser scanning microscopy (CLSM).

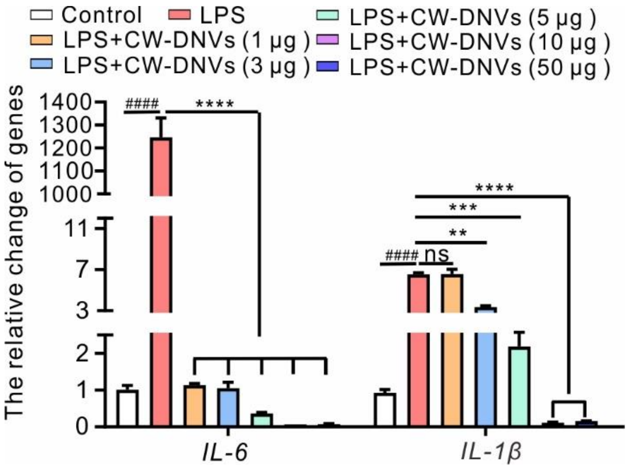

Supplementary Figure 5. Raw264.7 cells were treated with varying concentrations of CW-DNVs (1–50 μg total protein) for 24 h, followed by stimulation with LPS (50 ng/mL) for 4 h. The mRNA levels of *IL-6* and *IL-1β* were assessed by qPCR. Data are presented as mean ± SEM ($n = 3$). One-way ANOVA with Tukey's multiple-comparison test. ns, not significant, $^{\#\#\#\#}P < 0.0001$ *vs.* control; $^{**}P < 0.01$, $^{***}P < 0.001$, $^{****}P < 0.0001$ *vs.* LPS only.

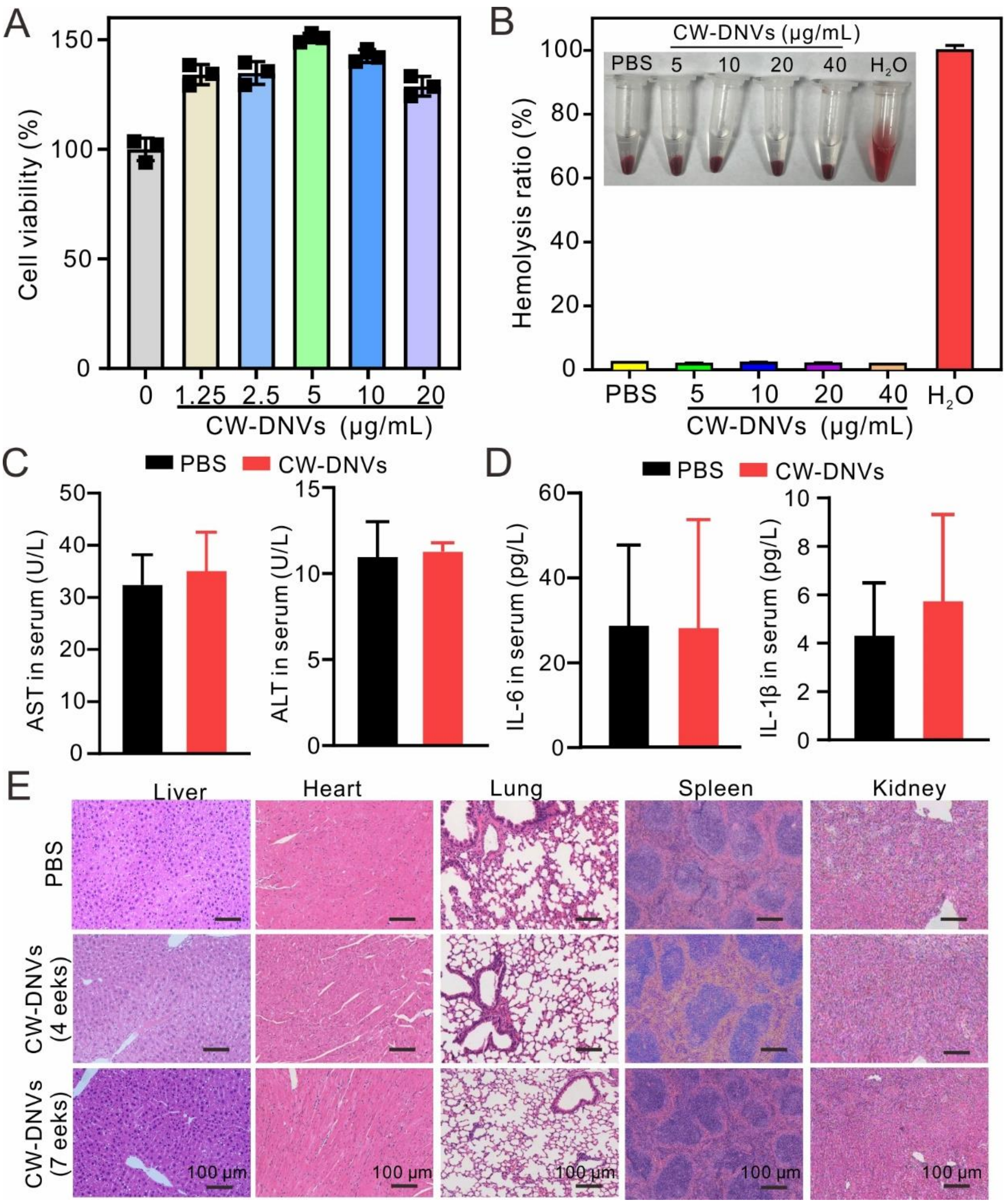

Supplementary Figure 6. Biosafety assessment of CW-DNVs. (A) Raw264.7 cell viability after 24 h exposure to graded CW-DNVs doses (MTT assay); (B) Hemolysis induced by CW-DNVs at 0-40 μg/mL; PBS and distilled water served as positive and negative controls, respectively; (C and D) Serum AST/ALT activities (C) and IL-6/IL-1β concentrations (D) in mice receiving 4 weekly injections of 150 μg CW-DNVs; (E) Representative HE-stained sections of liver, heart, lung, spleen, and kidney in mice receiving 4 and 7 weekly injections of 150 μg CW-DNVs. Data are presented as mean ± SEM (*n* = 3).

Supplemmentary Table 4. Primer sequences in this study

Gene name	Primer sequences
Mus-*Gapdh*	F 5′-GGGTTCCTATAAATACGGACTGC-3′
	R 5′- TACGGCCAAATCCGTTCACA -3′
Mus-*Il-6*	F 5′-CGGCCTTCCCTACTTCACAA-3′
	R 5′-TTGCCATTGCACAACTCTTTTC-3′
Mus-*Il-1β*	F 5′-GCAACTGTTCCTGAACTCAACT-3′
	R 5′-ATCTTTTGGGGTCCGTCAACT-3′
Mus-*Tnf-α*	F 5′-GACGTGGAACTGGCAGAAGAG-3′
	R 5′-TTGGTGGTTTGTGAGTGTGAG-3′
